# Management of primary sclerosing cholangitis: Current state-of-the-art

**DOI:** 10.1097/HC9.0000000000000590

**Published:** 2024-11-15

**Authors:** Guilherme Grossi Lopes Cançado, Gideon M. Hirschfield

**Affiliations:** Division of Gastroenterology and Hepatology, Department of Medicine, The Autoimmune and Rare Liver Disease Programme, Toronto General Hospital, University Health Network, Toronto, Ontario, Canada

**Keywords:** cholangiocarcinoma, colorectal cancer, disease management, dominant stricture, ursodeoxycholic acid

## Abstract

Primary sclerosing cholangitis is a chronic liver disease characterized by progressive inflammation and fibrosis of medium-large bile ducts, most commonly in association with inflammatory bowel disease. Most patients have a progressive disease course, alongside a heightened risk of hepatobiliary and colorectal cancer. Medical therapies are lacking, and this, in part, reflects a poor grasp of disease biology. As a result, current management is largely supportive, with liver transplantation an effective life-prolonging intervention when needed, but not one that cures disease. Emerging therapies targeting disease progression, as well as symptoms such as pruritus, continue to be explored. The trial design is increasingly cognizant of the application of thoughtful inclusion criteria, as well as better endpoints aimed at using surrogates of disease that can identify treatment benefits early. This is hoped to facilitate much-needed advances toward developing safe and effective interventions for patients.

## Graphical abstract

**Figure:**
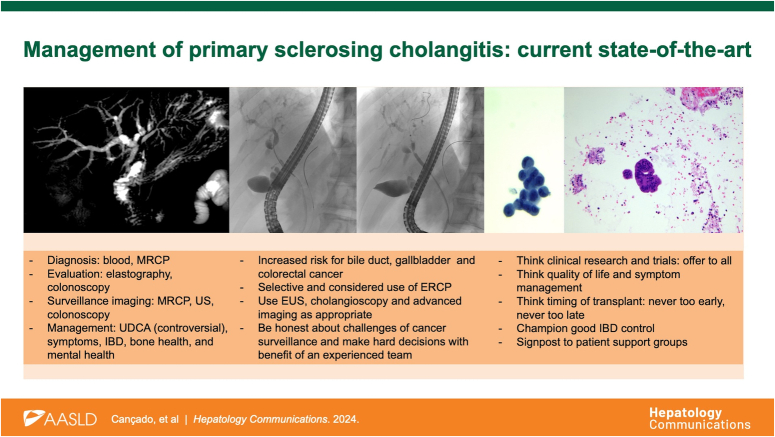

## INTRODUCTION

Primary sclerosing cholangitis (PSC) is an impactful disease for patients and a challenging disease for hepatology providers from the point of initial diagnosis, through to clinical crunch points such as timing of liver transplantation (LT), managing colonic or biliary dysplasia, as well as helping patients overcome debilitating symptoms such as pruritus.^1^ As a rare fibrosing inflammatory cholangiopathy of unknown etiology, stricturing of intrahepatic and/or extrahepatic bile ducts leads to progressive biliary disease as well as a marked risk of hepatobiliary malignancy.^1^,^2^,^3^ Depending on patient sex and heritage, the disease is commonly associated with inflammatory bowel disease (IBD), more usually of ulcerative colitis–like phenotype, but with a much higher occurrence of colon cancer.^4^ Most patients have multifocal strictures and dilatations of large bile ducts at cholangiography. A minority (5%–10%) may present with a variant called small-duct PSC, characterized by an initial normal biliary tree on cholangiogram, cholestatic liver biochemistry, histological features of biliary disease, and an absence of genetic or alternative causes of cholestasis.^5^ A variant with more pronounced hepatitic features akin to autoimmune hepatitis is also seen in perhaps 10% of patients, more commonly so for those presenting in childhood.^6^

## EPIDEMIOLOGY

PSC is rare, and the literature cites various low incidence and prevalence figures with geographical variation, and a greater challenge to define epidemiology trends for patients without IBD.^7^,^8^,^9^ In Ontario, Canada, for example, for every 50 people developing IBD annually, 1 person develops PSC annually, and for every 100 people with IBD, 1 will be living with PSC-IBD currently.^10^ Bimodal presentations are recognized, with a mean age of 15 years for childhood-onset disease, and 35 years for adults.^7^ Classically, men are more frequently affected than women, and ~50%–70% of patients have concurrent IBD, mainly ulcerative colitis.^11^,^12^ Among individuals with PSC-IBD, patients who received the diagnosis of PSC at an age younger than 40 years, men, and individuals of African Caribbean heritage are reported as having an increased incidence of PSC-related events.^13^ Other immune-mediated diseases are observed more frequently, such as type 1 diabetes, celiac disease, or sarcoidosis.^14^ People living with PSC have twice the overall risk of developing cancer compared to the general population, especially colon, bile duct, and gallbladder malignancies, and a 4-fold increase in mortality.^15^,^16^ Hepatopancreatobiliary cancers develop most often in association with classical PSC, with only a small number of such events occurring in patients with small-duct PSC.^17^,^18^,^19^ Overall, cholangiocarcinoma (CCA) and colorectal cancer account for ~32% and 8% of PSC-related mortality, respectively, compared to 15% for liver failure and 9% for liver transplant–related complications.^8^

## CLINICAL PRESENTATION, DIAGNOSIS, AND DIFFERENTIALS

The natural history of PSC is variable; large-scale surveys demonstrate differences in the course of disease based on whether ascertainment is through secondary or tertiary care.^20^ Currently, most patients are asymptomatic at the time of diagnosis and discover the disease after liver tests are found to be abnormal, either noted as part of their IBD care or incidentally.^21^ Several signs and symptoms, including pruritus, jaundice, fatigue, sleep disturbances, weight loss, urine/stool color changes, and abdominal pain, can develop as the disease progresses and fluctuates in severity.^22^,^23^ Patients with advanced PSC frequently develop decompensated cirrhosis and its complications. Bacterial cholangitis may complicate stricturing disease; presentation is different from classical cholangitis, and the triad of fever, jaundice, and pain are less overt because the disease is chronic and bacterial colonization is not acute.^24^ Patients also have a tendency to gallbladder dysfunction (eg, increased fasting and postprandial gallbladder volume), gallbladder stones, and a frequent need for cholecystectomy.^25^,^26^,^27^ In PSC, an enlarged gallbladder has been associated with lower ALP values, while patients who undergo cholecystectomy have been reported to exhibit more severe cholangiographic features and elevated AST. It is speculated that the gallbladder may play a protective role in limiting bile acid cytotoxicity, through alleviation of hyper-pressure caused by biliary tract obstruction, modulation of the enterohepatic circulation, as well as bile acid pool composition.^28^ Osteopenia and osteoporosis can also be found later in the disease course, especially in patients with concomitant IBD.^2^,^29^

Akin to other cholestatic diseases, greater elevations in ALP or bilirubin over the course of the disease indicate a worse prognosis. However, unlike primary biliary cholangitis, fluctuations in serum liver tests are much more common, reflecting the variable bile duct obstruction caused by strictures, stones, and sludge.^21^,^30^,^31^ Markedly elevated aminotransferase activity is seen periodically, and while it should lead to consideration for a variant of autoimmune hepatitis, in clinical practice, it reflects fluctuating cholestatic liver injury and presumably different degrees of intermittent biliary obstruction.^32^,^33^ Elevated concentrations of immunoglobulin G and perinuclear ANCA positivity are observed but are not specific.^34^ About 10%–15% of patients with PSC have elevated serum IgG4 levels, without IgG4-related disease, and this finding may be associated with worse outcomes.^35^,^36^,^37^

Invasive cholangiography by endoscopic retrograde cholangiopancreatography (ERCP) is reserved for intervention or assessment of strictures for cancer and should not be considered a routine diagnostic modality. A high-quality MRI with cholangiopancreatography (MRCP) is usually diagnostic.^2^,^38^ Main findings include multifocal stricturing of intrahepatic and extrahepatic bile ducts with intervening segments that are relatively normal in caliber or mildly dilated (“beaded” appearance).^2^,^39^ Intraductal stones can be evident. Secondary causes of sclerosing cholangitis should be evaluated through a detailed clinical history (Table 1), and there are usually clear clinical indicators that a secondary etiology is likely.

**TABLE 1:** Secondary causes of sclerosing cholangitis

Mechanism	Disease
Obstruction	Choledocholithiasis
	Congenital (choledochal cysts, Caroli disease, and biliary atresia)
	Cholangiocarcinoma
Immune-mediated	IgG4-related sclerosing cholangitis
	Hepatic sarcoidosis
	Langerhans cell histiocytosis
	Mast-cell cholangiopathy
	Eosinophilic cholangitis
	Antibody-mediated rejection (liver allograft)
Infectious	Helminth infection (eg, *Clonorchis*, *Opisthorchis*, *Ascaris)*
	“AIDS-related” cholangiopathy
	Covid 19–associated sclerosing cholangitis
	Recurrent pyogenic cholangitis
	Cytomegalovirus infection
Ischemic	Hepatic artery thrombosis (eg, after liver transplantation)
	Systemic vasculitis
	Chronic portal vein thrombosis/portal biliopathy
	Non-anastomotic strictures after liver transplantation
	Transarterial chemotherapy/embolization therapy
	Sclerosing cholangitis of the critically ill patient
Toxic	Ketamine-induced sclerosing cholangitis
Iatrogenic	Surgical trauma
Genetic	Cystic fibrosis
	ABCB4 deficiency with low-phospholipid–associated cholelithiasis and intrahepatic stones

A liver biopsy is not generally needed but has a role if cholangiography is normal (eg, suspected small-duct PSC), if there is a concern for alternate liver disease, if corticosteroid-responsive immune-mediated hepatitis is suggested by serum liver tests, and sometimes as a component of clinical trials.^2^ The hallmark of PSC on histological assessment is concentric “onion skin” periductal fibrosis, but this may not always be evident given sampling error, and the peripheral nature of percutaneous liver biopsies.^40^ Other features include nonspecific bile duct proliferation, periportal inflammation, cholestasis, ductopenia, and varying degrees of fibrosis, which can be quantified by a scoring system such as the Nakanuma criteria.^41^,^42^ Patients with normal MRI imaging but with typical histologic findings of sclerosing cholangitis, in the absence of IBD, should be tested for inherited forms of cholestasis (genetic cholestasis), before being diagnosed with small-duct PSC.^2^ The rate at which those with a small-duct variant progress to large-duct disease is cited as 14%–23% over 6–7 years, but this is more nuanced, as clinicians should be discouraged from using the term in young adults, in whom it is just more likely that, if diagnosed at an early stage, large-duct changes may not yet be evident.^18^,^19^

Finally, those who are not known already to have IBD should have a colonoscopy with random biopsies at PSC diagnosis to assess for concomitant IBD. In patients with PSC, subclinical histologic signs of IBD, including premalignant changes, may precede the development of clinical symptoms of IBD by several years.^43^ If IBD is not detected during the initial examination, it is appropriate to repeat the colonoscopy with biopsies at least once 5 years later or if there is new clinical suspicion of IBD. Later presentation of IBD, even after LT is also well recognized.

## RISK STRATIFICATION AND PROGNOSTIC SCORES

After diagnosis, risk stratification and prognostication are performed serially over time so that patients can understand their short-term, medium-term, and long-term prognosis. In most series, the average age for LT listing is ~48 years for people with PSC, and this is a useful parameter to remember for adult patients, especially those presenting classically with large-duct PSC-IBD and an elevated ALP.^44^ Clearly, ALP has been shown to be prognostic,^30^,^45^,^46^ but short-term variations were not associated with disease progression over a 2-year period in a trial setting.^31^

Liver stiffness measurement (LSM) by transient elastography (TE) or magnetic resonance elastography (MRE) can be used to estimate liver fibrosis. A French cohort reported a cutoff value by TE of 14.4 kPa for stage 4 fibrosis, with 0.88 diagnostic accuracy, while an annual increase of more than 1.3 kPa/y was associated with reduced transplant-free survival.^47^ On the other hand, an LSM by MRE >4.32 kPa and an increase above 0.34 kPa/y were linked to a significant risk of hepatic decompensation.^48^ MRE has been shown to have a higher correlation with Mayo Risk Score than TE.^49^

A score derived from MRI with MRCP findings (ANALI score) was also developed to predict radiologic progression (Table 2).^51^ Later, it was further validated to identify patients’ survival without LT or cirrhosis decompensation with a c-statistic of 0.89 (95% CI: 0.84–0.95) and 0.75 (95% CI: 0.64–0.87).^50^ A combination of an MRI score (ANALI score without gadolinium) with TE allowed the stratification of patients with PSC in low-risk (ANALI score ≤2 and LSM ≤10.5 kPa), medium-risk (ANALI score >2 or LSM >10.5 kPa), and high-risk (ANALI score >2 and LSM >10.5 kPa) groups for developing adverse outcomes. The 5-year cumulative rates of adverse outcomes in these 3 groups were 8%, 16%, and 38%, respectively.^52^ Unfortunately, the ANALI score is limited by poor-moderate interobserver agreement (intraclass correlation coefficient: 0.56, 95% CI: 0.42–0.68 without gadolinium), restricting its use in clinical practice.^53^ A 5-year longitudinal study demonstrated that the Enhanced Liver Fibrosis (ELF) test is more stable than the LSM measurement and is likely to perform better for risk stratification in PSC using single measurements.^54^ That said, ELF testing requires further evaluation in large cohorts; in a trial setting, change in ELF over time has been shown to predict clinical events.^55^ Recently, a quantitative MRCP tool using metrics calculated by postprocessing software has been developed (MRCP+).^56^ Ongoing evaluation is needed to understand the added value (as compared to serum liver tests, Fibroscan, MRE, existing MRI scoring systems, or ELF, for example) of capturing and tracking over time, such as quantitative biliary measures in routine and clinical trial practice.

**TABLE 2:** ANALI score—a combination of MRI features without and with i.v. gadolinium administration to predict the prognosis of patients with PSC

ANALI without gadolinium = (1× dilatation of intrahepatic bile ducts) + (2× dysmorphy) + (1× portal hypertension)	
ANALI with gadolinium = (1× dysmorphy) + (1× parenchymal enhancement heterogeneity)	
	Score
**Intrahepatic bile duct dilatation (mm)**	
≤3	0
4	1
≥5	2
Dysmorphy	
Absence	0
Presence	1
Portal hypertension	
Absence	0
Presence	1
Parenchymal heterogeneity	
Absence	0
Presence	1

*Note*: This score predicts event-free survival (liver transplantation and hepatic decompensation); score >2 without gadolinium: HR 24.95 (3.25–191.75); score >1 with gadolinium: HR 13.72 (1.76–107.03).^50^

Abbreviation: PSC, primary sclerosing cholangitis.

Different noninvasive predictive prognostic scores have been developed to risk stratify PSC^57^,^58^,^59^,^60^,^61^,^62^; these scores are summarized in Table 3 and can provide additional insights into disease-specific outcomes, particularly in advance of liver failure.

**TABLE 3:** Prognostic scores for PSC

Prognostic score	Variables included	Goal
Adult		
Revised Mayo risk score	Age, bilirubin, albumin, AST, history of variceal bleeding	Estimates overall survival up to 4 y of follow-up.
Amsterdam-Oxford model	PSC subtype, bilirubin, albumin, ALP, AST, platelets, age at PSC diagnosis	Estimates transplant-free survival at 5, 10, and 15 y of follow-up. C-statistic = 0.68
UK-PSC score	Age at diagnosis, bilirubin at diagnosis, albumin at diagnosis, platelets at diagnosis, hemoglobin at diagnosis, ALP at year 2, disease type (presence of extrahepatic disease), history of variceal bleeding by year 2	Estimates transplant-free survival at 2 and 10 y of follow-up. C-statistic = 0.80
PSC risk estimate tool (PREsTo)	Age, ALP, AST, bilirubin, albumin, sodium, hemoglobin, platelets, number of years since PSC was diagnosed	Predicts 5-y probability of hepatic decompensation. C-statistic = 0.90
Pediatric		
Sclerosing Cholangitis Outcomes in Pediatrics (SCOPE) Index	Bilirubin, albumin, platelet count, GGT, cholangiography (normal or large-duct involvement)	Predicts LT or death (TD) and hepatobiliary complications (HBCs = portal hypertensive, biliary, and cancer complications) within 5 y of follow-up. Validated for patients <18 years of age. C-statistics = 0.82 for TD and 0.76 for HBCs
Radiology		
DiStrict score	MRCP-defined ductal stricture with or without upstream dilatation and extent of ductal involvement	Predicts survival without liver-related outcomes (LT and liver-related death) through MRCP result. C-statistic = 0.78
qMRCP-PSC score	Artificial intelligence-driven analysis of MRCP-3D images	Predicts hepatobiliary complications, defined as the earliest occurring event among the following: (1) listing for LT; (2) liver-related death; (3) diagnosis of portal hypertensive complications such as ascites requiring hospital admission or diuretic therapy and HE requiring hospital admission; (4) gastroesophageal bleeding; (5) diagnosis of biliary complications (biliary strictures requiring dilation, stenting or external drainage, or hospitalization for acute bacterial cholangitis); (6) diagnosis of cholangiocarcinoma. C-statistic = 0.80

*Note*: C-statistics for risk scores are provided as per original studies. A C-statistic of 0.5 means that there is no predictive power at all, and 1.0 means perfect prediction of the outcome.

Abbreviations: HBC, hepatobiliary complications; LT, liver transplantation; MRCP, magnetic resonance cholangiopancreatography; PSC, primary sclerosing cholangitis; TD, liver transplantation or death.

## CLINICAL CARE

**FIGURE 1:**
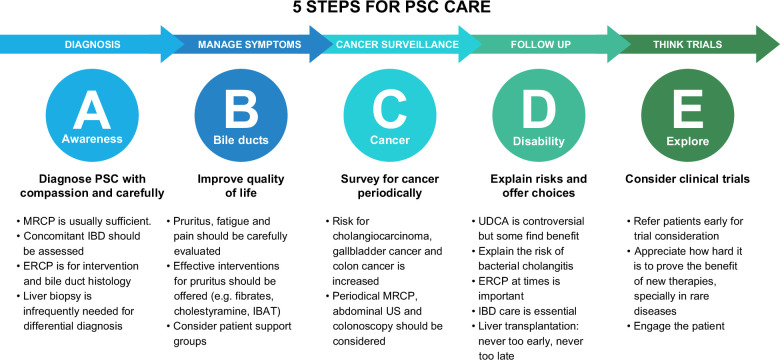
Five steps for PSC holistic care. Abbreviation: PSC, primary sclerosing cholangitis.

Effective disease-modifying options are lacking for people living with PSC. Ideally, the goals of care in PSC would focus on changing the natural history of disease (progression to end-stage liver disease and need for transplant and cancer development) and improving patient quality of life (Figure 1). Participation in clinical trials and research should be a priority for all if progress is to be made. Patient support groups are also a valuable source of support for patients.

### Ursodeoxycholic acid

Ursodeoxycholic acid (UDCA), a secondary bile acid generated in the colon, is a hydrophilic epimer of chenodeoxycholate and has been tested at different doses for the treatment of PSC. Although safe at doses <25 mg/kg/d, the benefit of UDCA in PSC is questionable.^63^,^64^ Several randomized controlled trials have been performed, but most of them were underpowered for the identification of relevant long-term clinical outcomes due to the small number of participants and short-term follow-ups. Meta-analyses demonstrate that UDCA has no beneficial effect on patients’ survival, liver histology, prevention of CCA, or improvement of clinical symptoms.^65^ UDCA treatment has been associated with improvement of serum markers of cholestasis, while UDCA discontinuation can lead to deterioration in symptoms, serum liver enzymes, and Mayo Risk Score.^66^ Furthermore, some studies suggested that patients with PSC who achieve normalization of elevated serum ALP levels may have an improved prognosis.^67^ In children, a 75% reduction in GGT or a GGT <50 IU was followed by better outcomes, regardless of UDCA treatment status, although lower GGT values were seen in treated (vs. untreated) patients.^68^ The most recent consensus advice recommends that patients who are not eligible or interested in clinical trials and who have increased ALP/GGT levels can be offered a trial with low to moderate doses of UDCA (13–23 mg/kg/d), which can be continued if there is meaningful reduction or normalization of ALP/GGT level, or improvement of symptoms, within 12 months.^2^,^69^ On the contrary, high-dose UDCA treatment (>28 mg/kg/d) was associated with worse disease outcomes and should be avoided.^64^ Overall, there is insufficient evidence for a beneficial effect of UDCA in reducing the risk of CCA and colorectal cancer, with no placebo-controlled trials addressing these questions, and a lack of robust data to support use for this context.^70^,^71^,^72^,^73^,^74^,^75^ In patients with PSC and ulcerative colitis, high-dose UDCA (28–30 mg/kg/d) was associated with an increased risk of colorectal neoplasia (dysplasia and cancer) in a randomized, placebo-controlled trial.^76^

### IBD

Several pathophysiological mechanisms have been proposed to explain the connection between the gut and liver in PSC, including dysbiosis, increased intestinal permeability, altered bile acid signaling, and aberrant lymphocyte homing.^38^ Interestingly, despite its close association, PSC has limited genetic correlation with IBD.^77^ Despite the close clinical association between PSC and IBD, when genome-wide studies are analyzed, the genetic correlation between PSC and IBD is less than seen clinically, supporting PSC-IBD to be distinct from IBD alone, for example, while the genetic architecture of PSC susceptibility is correlated with IBD, the whole genome correlations (r_g) is 0.46 for IBD, 0.62 for ulcerative colitis, and 0.24 for Crohn’s disease. IBD phenotype in terms of disease location and activity in patients with PSC seems different compared to classic ulcerative colitis or Crohn's disease. Colitis in PSC-IBD typically presents with extensive inflammation, but usually with milder activity. Pancolitis with or without backwash ileitis is common, inflammation is most active in the proximal colon, some areas of the mucosa may remain unaffected, and the rectum is relatively spared.^38^ It is controversial whether tight control of IBD activity can reduce liver disease progression.^78^,^79^ However, since the combination of IBD and PSC is associated with a higher risk of colorectal cancer, it is reasonable to aim for close disease control, alongside enhanced surveillance for dysplasia.

### Modulation of gut microbiome

Given genomic and clinical data, it is believed that in individuals with a genetic predisposition, elements of the microbiome (bacteria and fungi) or metabolic byproducts can potentially initiate an overactive immune response directed to the biliary tree.^80^ Patients with PSC have marked dysbiosis of the intestinal flora compared with the healthy population, altering essential nutrients and bile acid metabolism.^81^,^82^,^83^ In this way, the modulation of the gut microbiome and the intestinal permeability with antibiotics or fecal microbiota transplantation has gained attention. Multiple antibiotics have been evaluated, including minocycline, metronidazole, vancomycin and rifaximin, with inconclusive results. A recent meta-analysis with 124 patients with PSC showed that antibiotic treatment is associated with a statistically significant reduction in ALP (−33.2%), Mayo risk score (−36.1%), and bilirubin (−28.8%), with the greatest ALP decrease observed for vancomycin (−65.6%) use.^84^ Several small studies have demonstrated a significant reduction in liver enzyme values and Mayo risk score at 12 weeks of treatment with vancomycin.^85^,^86^ However, a large multicenter retrospective study with propensity score matching of children with PSC did not improve outcomes in patients who received vancomycin or UDCA, compared to a strategy of observation only.^87^ Some studies show beneficial effects of oral vancomycin for PSC-associated IBD, with improvement of diarrhea and induction of clinical and endoscopic disease remission.^88^,^89^,^90^ Following the same rationale, fecal microbiota transplantation has also been evaluated in PSC. A pilot study of fecal microbiota transplantation (single donor) in 10 people with PSC and concomitant IBD showed safety, improved bacterial diversity, and ALP reduction (≥50%) in some patients but not the majority.^91^ A prospective study involving 15 patients with PSC-IBD tested the effects of an 8-week gluten-free diet. Although no clinical improvement was observed, biomarkers of intestinal inflammation, microbiota composition, and barrier function did improve.^92^

## SUPPORTIVE TREATMENTS AND SURVEILLANCE

### Relevant strictures

**FIGURE 2:**
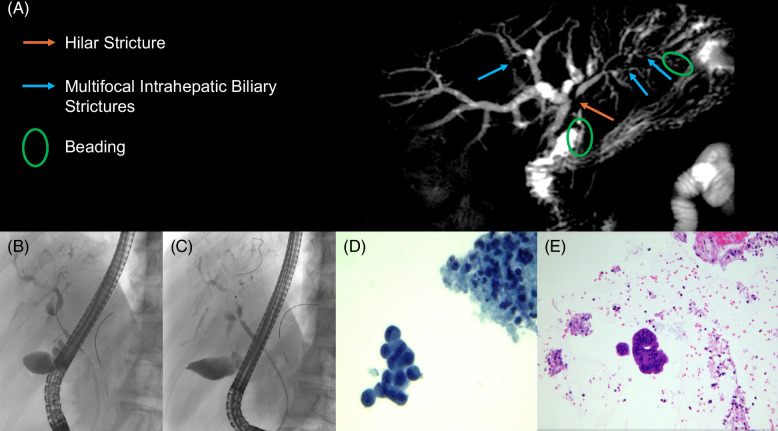
(A) Magnetic resonance cholangiopancreatography showing intrahepatic ductal dilatation with the beaded appearance of the intrahepatic ducts and common bile duct. (B) ERCP showing a complex hilar stricture, measuring about 3–5 mm in length, with upstream dilatation of intrahepatic ducts. (C) ERCP showing the stricture after dilation using a Hurricane balloon. (D, E) Cytology from brushing revealed a few groups of atypical epithelial cells in a background of inflammation, suspicious for malignancy. Abbreviation: ERCP, endoscopic retrograde cholangiopancreatography.

Strictures represent a fibrogenic response to persistent biliary inflammation (Figure 2). A dominant stricture has been defined as stenosis with a diameter of ≤1.5 mm in the common bile duct or ≤1 mm in the hepatic duct by ERCP. In clinical practice, the term relevant stricture is better used to refer to any biliary stricture of the common hepatic duct or hepatic ducts associated with signs or symptoms of obstructive cholestasis and/or bacterial cholangitis.^2^ The prevalence of dominant strictures is hard to really assess as patient cohorts are so variable, but when clearly present, can be associated with a higher risk of CCA and reduced transplant-free survival.^93^

Initially, noninvasive imaging for biliary obstruction or malignancy complication is usually with an MRCP, followed by other imaging modalities as guided by expert radiology review (eg, dynamic liver MRI, contrast computed tomography/ultrasound, and endoscopic ultrasound).^2^,^69^ When a progression of previously known strictures is demonstrated, or a totally new relevant stricture is identified, ERCP with ductal brush sampling and fluorescent in situ hybridization, if available, is recommended to rule out CCA. Biliary brush cytology alone lacks sensitivity (43%), despite having excellent specificity (97%) for the diagnosis of CCA.^94^ In case of indeterminate biliary strictures, fluoroscopically guided intraductal forceps biopsy, endoscopic ultrasound, intraductal ultrasonography, or even direct intraductal cholangioscopy with transpapillary bile duct biopsy are relevant tools to aid management.

Endoscopic treatment of clinically relevant strictures (eg, for jaundice, deterioration in serum liver tests, and clinical concern about new malignancy) is often needed in patients with PSC. The use of endoscopic intervention in other settings is generally avoided, given the risks of cholangitis and pancreatitis. The optimal endoscopic technique to properly address a biliary stricture is unclear, and ERCP expertise is probably more relevant. Although ERCP clearly carries risk, equally, PSC is a disease of biliary obstruction, and there is debate in the literature about whether the more intensive and regular endoscopic intervention could delay LT.^95^ However, widespread applicability to nonexpert programs is of concern, as well as challenges in comparing outcomes from very different clinical practice environments. In a multicenter randomized trial of patients with PSC and dominant stricture, balloon dilation presented similar efficacy to stent placement, but lower incidence of serious adverse events (pancreatitis and bacterial cholangitis).^96^ Nonetheless, there will be instances where a stent (plastic or metal) can be helpful. If cannulation is difficult, biliary sphincterotomy is advised, bearing in mind that these patients are likely to require multiple procedures throughout life and present various episodes of cholangitis. Biliary sphincterotomy was independently associated with an increased risk of short-term adverse events, while previous biliary papillotomy/sphincterotomy was protective for post-ERCP pancreatitis in subsequent ERCPs.^97^,^98^ The administration of rectal nonsteroidal anti-inflammatory drugs (100 mg of diclofenac or indomethacin), lactated ringer solution, and prophylactic antibiotics are recommended before ERCP to reduce procedure-related complications.^99^,^100^,^101^

## CHOLANGITIS

Approximately 6% of patients with PSC have bacterial cholangitis at diagnosis, and nearly 40% experience this complication over time.^2^ Patients with PSC with dominant strictures or previously submitted to ERCP, with or without therapeutic intervention, are at a higher risk of developing bacterial cholangitis, especially when stents are left in situ.^102^,^103^ The management depends on the severity of cholangitis, which can range from a subclinical to a severe infection. Biliary infections are often polymicrobial, but the most common organisms are *Escherichia coli*, *Klebsiella*, *Enterococcus*, *Clostridium*, *Streptococcus*, *Pseudomonas*, and *Bacteroides* species. In toxemic patients, hospitalization is necessary for blood culture, i.v. broad-spectrum antibiotic treatment, and hemodynamic support.^104^ Antifungal therapy should be considered in those not responding to the initial treatment, especially in persistently jaundiced patients. Prior work has shown that persistent *Candida* infection in bile is associated with reduced survival.^105^ Patients with severe acute cholangitis and dominant bile duct strictures require biliary decompression by therapeutic ERCP or percutaneous drainage, as the mortality in those untreated is high.^106^ In patients with recurrent bacterial cholangitis, long-term prophylactic rotating antibiotic therapy may be needed, while LT is also an option.^106^

## Cholangiocarcinoma

The lifetime risk of CCA in patients with PSC has been estimated to be up to 20%, while the annual incidence ranges from 0.6% to 1.5%.^106^,^107^,^108^,^109^ CCA is usually diagnosed in the fourth decade, in contrast to sporadic CCA, and hilar localization is most common. About one-third of CCAs are present in the first year after a diagnosis of PSC.^110^ Rapid worsening of liver tests or clinical deterioration during follow-up should raise a concern about CCA or dominant strictures, which are the most frequent complications of PSC and should trigger a new imaging investigation (Figure 2).^111^ Predictors of CCA in PSC remain unclear. Advanced age, dominant stricture, bile duct diameter differences >5.1 mm, male sex, IBD and PSC duration, elevated bilirubin levels, and tobacco and alcohol consumption have been described as risk factors for CCA in PSC.^2^,^11^,^112^ Detecting CCA in PSC is challenging, partly due to its insidious presentation, overlapping findings with bile duct stricture progression, and low sensitivity of bile duct brushings and biopsy for its diagnosis. On the other hand, timely detection of CCA is extremely important since it may be treatable at early stages. Clear evidence for the utility of MRCP surveillance in PSC is lacking and at the present time guidelines recommend at least annual imaging surveillance for patients with large-duct PSC who are more than 18 years of age, by MRI/MRCP (with abdominal ultrasound as an alternative); CA19-9 is considered optional.^2^,^69^ Increased CA 19-9 levels may sometimes support a diagnosis of CCA, but a normal level does not rule out a tumor.^113^,^114^ Notably, a significantly elevated CA 19-9 >1000 U/mL may indicate the presence of metastatic disease.^115^ It is, however, important to note that CA 19-9 levels may increase in cases of biliary obstruction and that ~10% of patients, particularly those with the Lewis^α-β-^ genotype, do not secrete CA 19-9.^116^ Recently, a multicenter study from Australia showed that MRCP surveillance is associated with 71% reduced risk of death (HR: 0.29, 95% CI: 0.14–0.59, *p* < 0.001) and increased likelihood of having earlier ERCP. However, survival after hepatobiliary cancer diagnosis was not significantly different between patients submitted or not to MRCP surveillance.^117^ In contrast, a Swedish prospective study with 512 patients with PSC showed that annual imaging and tumor marker surveillance were not associated with improved cancer-related survival.^118^ Finally, Trivedi et al^13^ demonstrated that among patients with PSC-IBD, annual imaging might be beneficial, but after the exclusion of all CCA cases from the first year, there was no difference in post-CCA survivorship between the regular surveillance versus no surveillance groups.

## GALLBLADDER POLYPS AND CANCER

The lifetime frequency of gallbladder cancer in PSC ranges from 0.6% to 3.5%, while gallbladder polyps are found in 10%–17% of patients.^119^ A large retrospective cohort showed that gallbladder cancer is associated with polyps >10 mm and interval growth or mass-like lesions on imaging.^120^ Evidence from previous studies supports the cutoff value at a polyp size of 0.8 cm with a sensitivity of 97% and a specificity of 53% for neoplasia.^121^ Overall, gallbladder carcinoma surveillance should be performed at least annually, preferably by abdominal ultrasound. People with PSC and gallbladder polyps ≥8 mm or smaller growing-in-size polyps should be offered a cholecystectomy, but the decision should always take into consideration the underlying liver function and the risk of hepatic decompensation. Polyps ≤8 mm may be monitored with abdominal ultrasound every 6 months.

## COLORECTAL CANCER

There is a well-established increased risk for colorectal cancer with concomitant PSC and IBD colitis. In a meta-analysis of 116 studies, the prevalence of colorectal cancer was 3.7%, with cumulative risks of 2%, 8%, and 18% at 10, 20, and 30 years, respectively.^122^ Even after LT, an incidence of colorectal cancer of 1% per person per year has been reported in patients with PSC and ulcerative colitis.^123^ Compared with patients with PSC-only, patients with PSC-IBD association are more likely to be diagnosed with colorectal cancer (23.3% vs. 1.8%, *p* < 0.01) and either low-grade or uncharacterized dysplasia (16.7% vs. 0.0%, *p* < 0.01).^124^ In this way, an annual or biannual high-definition surveillance colonoscopy with multiple targeted and random biopsies should be recommended from the time of diagnosis for all patients with PSC (including post-orthotopic LT) and IBD. Advanced surveillance should be used ideally by endoscopists with appropriate expertise. Colectomy is generally indicated for patients with high-grade dysplasia and multifocal low-grade dysplasia.^2^,^69^ Unfortunately, a common complication of patients with PSC-IBD submitted to total proctocolectomy with ileal pouch-anal anastomosis is chronic pouchitis, which is known to be less responsive to conventional antimicrobial therapy than among patients without PSC.^125^

## Hepatocellular Carcinoma

Cirrhosis is the most important risk factor for HCC in patients with PSC. One large study reported an HCC prevalence of ~2% during nearly 10 years of follow-up.^126^ Patients with PSC cirrhosis should undergo HCC surveillance with an abdominal ultrasound every 6 months.

## PRURITUS AND QUALITY OF LIFE IN PSC

Data regarding health-related quality of life and patient-reported outcomes in PSC is relatively limited. Twenty to 60% of patients with PSC suffer from pruritus, which can be severe and lead to social isolation, sleep disturbances, and depression.^2^,^127^,^128^ A recent longitudinal study from the EpiPSC2 study group showed a small significant reduction in several health-related quality of life scores compared with a Dutch reference population, describing a negative association with female sex, older age, advanced disease stage, IBD status, and presence of itching.^129^ Current treatment options for the management of cholestatic pruritus are limited and mostly rely on cholestyramine 4–16 g/d. Off-label options include fibrates (bezafibrate 400 mg/d), rifampicin (150–300 mg/d), opioid antagonists (naltrexone 50–100 mg/d), and sertraline (50 mg/d). In a short randomized controlled trial (FITCH trial), a reduction of at least 50% of pruritus intensity, as determined by the visual analog scale score, was reached by 41% of patients with PSC treated with bezafibrate compared with 11% of the placebo group.^130^ Recently, clinical research has focused on the potential of ileal bile acid transporter inhibitors to reduce pruritus (eg, NCT 04663308).

## Liver Transplantation

The lack of effective disease-modifying therapy frequently leads to PSC progression towards end-stage liver disease, requiring LT. Patients with PSC comprise 4%–5% of the transplant waitlist, are underprivileged by a MELD score organ allocation system, and frequently benefit from access to living donor LT if deceased organs are limited either by a lack of donors or insufficient use of organ reperfusion technologies.^9^,^131^ Other indications for LT in PSC are intractable pruritus, recurrent bacterial cholangitis, HCC, and early-stage CCA/high-grade biliary dysplasia, but the criteria vary between countries. Patient and graft survival after LT are comparable with those transplanted for other causes, with a 5-year survival >80%.^132^ The MELD score and its iterations (eg, MELD-Na and MELD 3.0) are currently used in most centers around the world to waitlist PSC for LT. However, it frequently does not capture the severity of the disease, in part due to underweighting of the bilirubin and liver-related complications, such as bacterial cholangitis and CCA.^133^ Although not without some controversy,^134^ outcomes for live donor transplants appear the same as with deceased donor transplantation.^131^,^135^ Patients with PSC with potential living donors on the waitlist have a 60% reduction in the risk of death compared to those without potential living donors.^135^ Recurrent PSC (rPSC) is reported to occur in 20%–30% of patients after LT.^136^ Living donor transplantation, even from first-degree relatives, does not increase the risk of rPSC. Male sex, younger age, extended criteria grafts, steroid-free anti-thymocyte globulin induction protocols, cholestasis within 3–12 months following LT, primary immunosuppression with tacrolimus, allograft rejection, and active IBD status have been described as risk factors for recurrence.^137^,^138^ The role of colectomy remains controversial as regards preventing recurrent disease, and the concern over enhanced risk for pouchitis (and its impact on graft outcome) in PSC-IBD is another area of controversy. rPSC is usually characterized by cholangiographic evidence of intrahepatic and/or extrahepatic multifocal biliary strictures occurring >90 days after LT, and/or histological evidence of fibrous cholangitis, fibro-obliterative ductular lesions, and ductopenia, in the absence of chronic allograft rejection, hepatic ischemia, or donor-recipient blood type incompatibility.^139^ People with rPSC should be treated like classical PSC, receive appropriate endoscopic therapy, and be considered for retransplantation if needed.^137^

## EXAMPLES OF TRIAL THERAPIES CONSIDERED

### nor-Ursodeoxycholic acid

24-norursodeoxycholic acid is a side chain–shortened C23 homolog of UDCA with anticholestatic, anti-inflammatory, and antifibrotic properties in animal models. A phase II study, which included 161 patients with PSC with elevated serum ALP levels, revealed up to 26% reduction in serum ALP level after 12 weeks of 24-norursodeoxycholic acid treatment, with a good safety profile.^140^ A phase III clinical trial (NCT03872921) is currently underway to compare 1500 mg/d of 24-norursodeoxycholic acid with a placebo for PSC treatment.

### Peroxisome proliferator–activated receptor agonists

The peroxisome proliferator–activated receptors play an important role in bile acid detoxification. Adjusting the composition of total bile acids to less toxic bile acid glucuronides is a relevant potential therapeutic approach. Among the many functions of the peroxisome proliferator–activated receptor pathways as regards bile acid biology, peroxisome proliferator–activated receptor α, in particular, also regulates uridine 5′-diphospho-glucuronosyltransferase enzymes, including those related to bilirubin and bile acid glucuronidation in the liver.^141^ Previous studies have shown that fibrates improve liver biochemistry and long-term prognosis in primary biliary cholangitis.^142^ A variety of small studies have been performed in PSC that point toward biochemical improvements, but definitive large, placebo-controlled trials, with appropriate follow-up, and endpoints beyond serum liver tests, remain needed.^143^,^144^,^145^,^146^

### Novel approaches and medications

Cenicriviroc, a dual C-C chemokine receptor types 2 and 5 antagonist, has been evaluated in a small exploratory study due to its potential anti-inflammatory and antifibrotic activity, and induced only a minor reduction in ALP (−18.0%) after 24 weeks of treatment.^147^ In a phase II trial, NGM282, a first-in-class, engineered analog of the endocrine hormone FGF19, inhibited bile acid synthesis and decreased fibrosis markers without significantly affecting ALP levels.^148^ On the other hand, obeticholic acid, a potent farnesoid X receptor agonist approved for the treatment of primary biliary cholangitis, reduced ALP in 14% to 30%, depending on the dose and concomitant use of UDCA in a randomized placebo-controlled, dose-finding, phase II PSC trial. A dose-related increase in pruritus score was observed in up to 67% of the patients, with 41% of those treated with OCA 5–10 mg presenting severe itching.^149^ Similarly, cilofexor, a nonsteroidal farnesoid X receptor agonist, induced a 21% reduction in ALP after 12 weeks in patients with PSC and elevated ALP and without cirrhosis, a benefit that was sustained in a 96-week extension trial. Severe pruritus was observed in only 9% of the patients.^150^ Most recently, a pre-planned, interim futility analysis of the phase III trial (PRIMIS), designed to assess the efficacy and safety of cilofexor in non-cirrhotic PSC, led to early termination of the trial due to a low probability (6.8%) of achieving its primary endpoint. Except for ALT levels, there were no notable changes from baseline to week 96 in markers of liver injury or fibrosis or PSC symptoms with cilofexor versus placebo.^151^ In the same direction, treatment with the Lysyl oxidase-like 2 inhibitor simtuzumab for 96 weeks did not provide clinical benefit in patients with PSC.^55^ The biologics vedolizumab, adalimumab, and infliximab, commonly used for IBD treatment, were assessed in the subgroup of patients with PSC-IBD but did not show convincing evidence for liver biochemical response.^152^ Recently, berberine ursodeoxycholate (HTD1801), ionic salt of berberine and UDCA, was associated with improvements in ALP, ALT, AST, and GGT, with a greater percentage of patients achieving clinically relevant ALP reduction thresholds (ALP <1.5 × upper limit of normal = 26%, at least 50% reduction in ALP = 17%, and normalization of ALP = 9%) than placebo.^153^ Finally, timolumab, a monoclonal antibody against vascular adhesion protein 1, did not show significant changes in serum liver tests in a phase II clinical trial including patients with PSC.^154^

Appropriate efforts are being made to improve drug development pathways in PSC that capture the combined challenges of studying a rare disease with marked heterogeneity alongside inadequately validated surrogates of outcome. Studies in the future will no doubt seek well-thought-out inclusion/exclusion criteria, clinically meaningful surrogates of outcomes, and enhanced definitions for important clinical endpoints such as cholangitis. Attention to alternate study designs that focus on greater use of long-term real-world data, as well as comparisons of treated populations with historical data, will also prove important.

## CONCLUSIONS

PSC remains a challenging disease for patients and their families, characterized by a poorly understood pathogenesis, a close but distinct relationship with IBD, and a variable but usually impactful clinical progression over a long time period. While our understanding of the immunological and genetic factors driving PSC has grown, translating these insights into tangible therapeutic breakthroughs remains elusive. The rarity of the disease and the need to address many facets of the disease (progression, symptoms, and cancer) coupled with the lack of clearly attainable but meaningful clinical trial endpoints further complicates the development of effective treatments. As a result, current management remains compassionate, supportive, tailored to the individual, and anchored in ensuring high-quality ambulatory hepatology care, timely access to transplantation, proactive care for symptoms, and appropriate intensity cancer surveillance. Collaborative global efforts, including patients, providers, drug regulators, and pharma, are essential to drive the discovery of effective therapies, which will ultimately lead to improving the prognosis and quality of life for individuals with PSC.
